# Case series: indoor-photosensitivity caused by fluorescent lamps in patients treated with vemurafenib for metastatic melanoma

**DOI:** 10.1186/1471-2407-14-967

**Published:** 2014-12-17

**Authors:** Steve Boudewijns, Winald R Gerritsen, Rutger H T Koornstra

**Affiliations:** Department of Medical Oncology, Radboud University Medical Center Nijmegen, PO Box 9101, 6500 HB Nijmegen, The Netherlands

**Keywords:** Melanoma, Vemurafenib, Fluorescent lamps, Indoor-photosensitivity

## Abstract

**Background:**

Vemurafenib, a selective inhibitor of genetically activated BRAF, is registered for unresectable stage III and stage IV melanomas harboring a BRAF mutation. Photosensitivity related to exposure to sunlight is a common side-effect. We here present three cases of indoor-photosensitivity due to fluorescent lamps, whilst undergoing treatment with vemurafenib.

**Case presentation:**

Patient A is a 45-year-old Caucasian female, patient B a 32-year-old Caucasian male and patient C a 53-year-old male. They are all undergoing treatment with vemurafenib for metastatic melanoma. Patient A developed indoor-photosensitivity due to fluorescent lamps at work. Her employer changed the lighting to LED light and her complaints disappeared. Patient B is a biology teacher and in classrooms he is exposed to fluorescent lamps. He developed alopecia and subsequently indoor-photosensitivity. This was solved by wearing a baseball cap at work during the day. Patient C developed red and burning skin after working under fluorescent lamps in his shed. This side-effect disappeared completely after avoiding the lamps.

**Conclusion:**

Photosensitivity is a known adverse event of vemurafenib. This is known to be an UVA-depended photosensitivity. Until now it was thought to be solely related to sunlight exposure. These cases illustrate that patients, whilst undergoing treatment with vemurafenib, can develop indoor-photosensitivity as a result of exposure to fluorescent lamps with a relatively high UV content of the emitted spectrum (low permissible exposure time). Awareness of this side-effect is important to take appropriate measures in the future.

## Background

Activating BRAF kinase mutations occur in approximately 60% of melanomas [1]. Vemurafenib is a selective inhibitor of genetically activated BRAF [2]. Vemurafenib is registered for patients with BRAF V600 mutation-positive unresectable stage III or metastatic melanoma. The best-known side effect of vemurafenib is cutaneous toxicity, e.g. rash, photosensitivity and squamoproliferative eruptions [3–6]. Until now photosensitivity has been related to sunlight exposure. Here we present, to our knowledge, the first cases of indoor-photosensitivity related to exposure to fluorescent lamps during vemurafenib use.

## Case presentation

Patient A is a 45-year-old Caucasian female who was referred to our hospital in June 2012 with metastatic melanoma (lungs, liver and lymph nodes). Mutation analysis of the BRAF gene showed a V600E mutation in exon 15. Her history revealed a nodular melanoma on her back in May 2010, Clark-level 3 and Breslow depth 0.9 mm. On June 6, 2012 the patient started with vemurafenib 960 mg bi-daily (BID). In October 2012 she noticed red and burning skin on her head and hands. She did not report any sun exposure. Therefore this was considered to be photosensitivity related to ultraviolet (UV) light exposure from fluorescent lamps at her workplace. At the moment she mentioned her complaints to her employer, he changed the fluorescent lamps to LED light, which immediately led to the complete disappearance of her complaints. Unfortunately, progression occurred three months later, in January 2013, and vemurafenib was discontinued. Consecutively she received dacarbazine 1000 mg/m^2^ and ipilimumab 3 mg/kg with limited effect and palliative radiotherapy was delivered because of metastases of the brain and right hip. In view of substantial progression without other potential treatment options vemurafenib was re-challenged in August 2013, but substantial clinical benefit did not come. Treatment with vemurafenib was stopped and patient died in October 2013.

Patient B is a 32-year-old Caucasian male with metastatic melanoma (lungs, lymph nodes, right adrenal gland), with unknown primary site, since November 2011. Mutation analysis of the BRAF gene showed a V600E mutation in exon 15. At first the patient was included in a trial with dendritic cell vaccination and cisplatin (50 mg/m^2^), but due to progression in May 2012, vemurafenib 960 mg BID was started. The dose was reduced to 720 mg BID due to grade 3 myalgia. After 3 months the patient developed vemurafenib-related alopecia and he suffered from a burning sensation of the scalp with erythema when working in the classroom (full time biology teacher at a high school). He did not experience any redness or itchiness when out of doors, but then he carefully applied sun block lotion. His side effects disappeared on wearing a baseball cap in the classroom. Based on these observations, it was concluded that photosensitivity was related to fluorescent lamps in his classroom. In March 2013 vemurafenib was switched to ipilimumab 3 mg/kg, because of disease progression. Unfortunately there was a rapid clinical progression and our patient died in May 2013.

**Figure 1:**
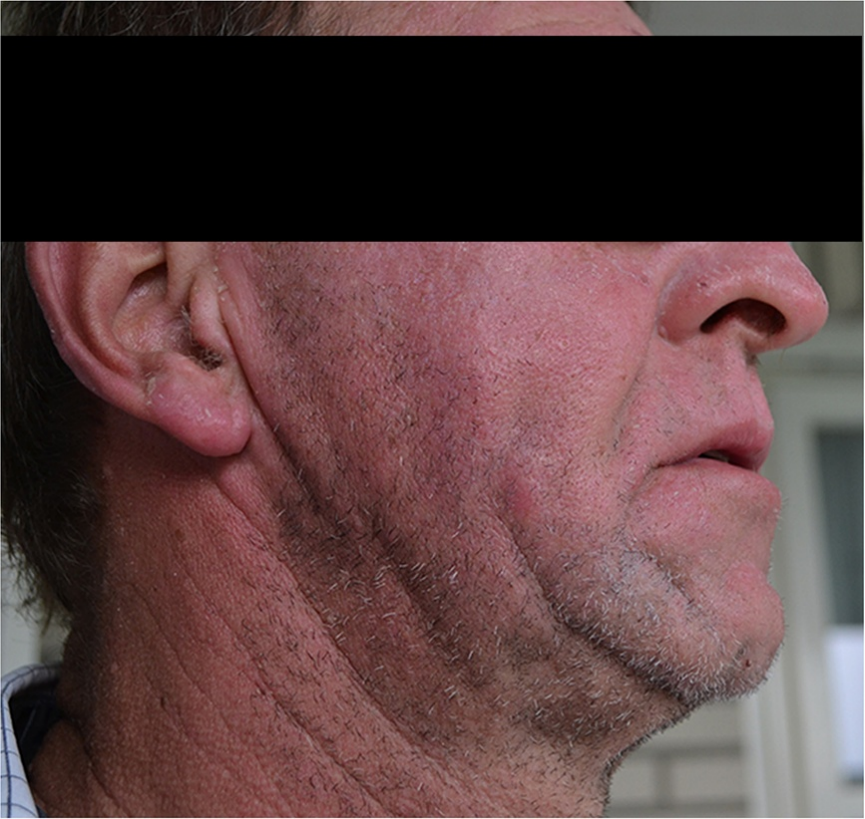
**Indoor-photosensitivity in patient C.**

Patient C is a 53 year-old Caucasian male who was referred to our hospital in April 2014 because of metastatic melanoma (liver, lymph nodes and skin). He started with vemurafenib 960 mg BID in May 2014. He soon noticed red and burning skin on his face (Figure 1) after working under fluorescent lamps (36 Watt lamps) for a few days in a row in his shed. This disappeared after a few days of avoiding the fluorescent lamps. At this moment the patient is still taking vemurafenib and he is in a good clinical condition.

## Conclusions

Photosensitivity is a known adverse event of vemurafenib, with up to 30% of treated patients in the phase III trial [3, 6]. All patients are therefore counseled before treatment with regards to sun exposure. Nevertheless photosensitivity is still a common adverse event [6]. Dummer showed a markedly reduced minimal erythema dose of ultraviolet A (UVA) light in patients using vemurafenib, while it was normal for ultraviolet B (UVB). They concluded that vemurafenib can induce an UVA-dependent phototoxicity [7]. In order to take appropriate measures it is also important to know the potential alternative sources of UV radiation. It has been reported earlier that fluorescent light can have an effect on photosensitive skin-related conditions, like polymorphic light eruption, chronic actinic dermatosis and lupus erythematosus [8].

In our three cases, the patients treated with vemurafenib suffered from photosensitivity while being exposed to regular fluorescent lamps. It is not widely known that the emitted spectrum of some fluorescent lamps contains UV radiation, which consists mainly of UVA [9].

The dermatological symptoms of all three patients disappeared from the moment they avoided exposure to the fluorescent lamps. Therefore we expected that the fluorescent lamp exposure was the most probable source of UV-radiation in our vemurafenib treated patients.

**Figure 2:**
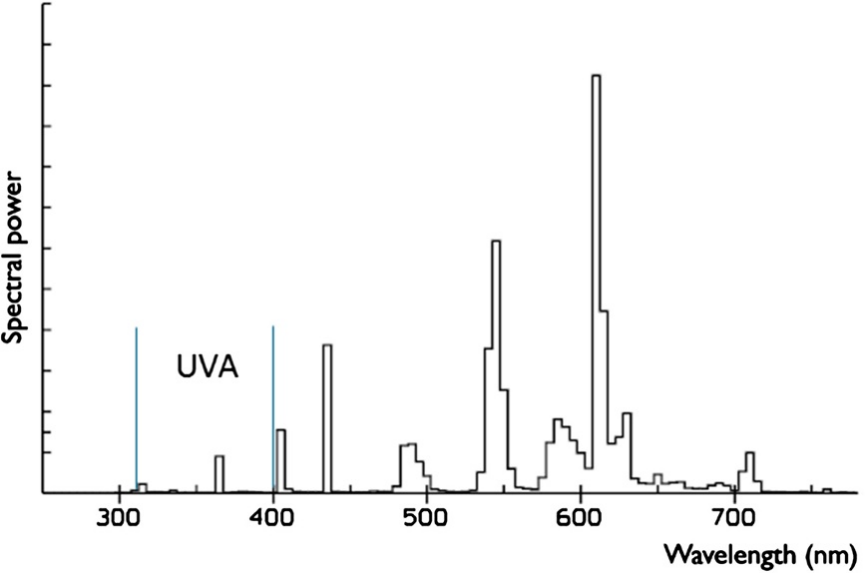
**Spectrum of a 36 W fluorescent lamp (colour 827) with PET 22 klx · h.** The permissible exposure time (PET) reflects the amount of UV radiation. (UVA: 315–400 nm, UVB 280–315 nm, UVC 100–280 nm) [9, 11].

It is difficult to exactly compare the amount of UVA in sunlight with the amount of UVA in fluorescent lamps, because of many variables (e.g. geographic region and current season). For example, a normal summer’s day in June in the Netherlands has an UV index of 6 milliWatt/m^2^ and a winter’s day in January in the Netherlands has an UV index of 0.5 milliWatt/m^2^. There are 36 Watt fluorescent lamps, with lamp color 827 with a permissible exposure time (PET) of 22 kilolux · hour (klx · h), which have an UV index of about 0.015 milliWatt/m^2^ (Figure 2). Eight hours under these fluorescent lamps compares with about 1.2 minutes in the sun during summertime, and with about 14 minutes in the winter [10]. Dummer showed that the minimal erythema dose is rapidly reduced when patients are using vemurafenib compared to non-users. Walking a short distance (few minutes) to a car without use of sun block lotion can already induce erythema in patients treated with vemurafenib. Taking all this information together, it is very likely that sitting under fluorescent lamps for a longer period of time can cause an UVA-dependent photosensitivity in patients treated with vemurafenib.

It is probable that not all fluorescent lamps will cause problems, while the UV content of the emitted spectrum depends on both the phosphor and the glass envelope of the fluorescent lamp [8]. If a lamp has a high PET, for instance >999 klx · h, then it has a much lower UV index. Eight hours under these lamps compares with about 1.5 seconds in the sun during the summer in the Netherlands.

These are the first known cases describing indoor-photosensitivity caused by fluorescent lamps in patients using vemurafenib. These three cases should not be the reason for all patients to avoid fluorescent light, however one should recognize this as a possible source of UVA exposure and patients should be counseled when suffering from symptoms of indoor-photosensitivity. These patients can be advised to avoid fluorescent lamps with a relatively high UV content of the emitted spectrum (low permissible exposure time). Alternatively other light sources can be applied, such as LED lighting, since this contains no UV radiation.

## Consent

Written informed consent was obtained from the patients or their family for publication of this Case report and any accompanying images. A copy of the written consent is available for review by the Editor of this journal.

## Abbreviations

BID: Bi-daily
klx · h: Kiliolux per hour
UV: Ultraviolet
UVA: Ultraviolet A
UVB: Ultraviolet B.
